# Impact of the Synthetic Strategy on the Structure and Availability of Active Sites in Bifunctional Mesoporous Organic–Inorganic Hybrids

**DOI:** 10.3390/ma18214937

**Published:** 2025-10-29

**Authors:** Julio Cesar Fernandes Pape Brito, Gioele Ancora, Ivana Miletto, Enrica Gianotti

**Affiliations:** 1 Department for Sustainable Development and Ecological Transition, Università del Piemonte Orientale, Piazza Sant’Eusebio 5, 13100 Vercelli, Italy; juliocesar.fernandes@uniupo.it; 2 Department of Science and Technological Innovation, Università del Piemonte Orientale, Viale Teresa Michel 11, 15100 Alessandria, Italy; gioele.ancora@uniupo.it; 3 Department of Pharmaceutical Sciences, Università del Piemonte Orientale, Largo Guido Donegani 2-3, 28100 Novara, Italy; ivana.miletto@uniupo.it

**Keywords:** bifunctional organic-inorganic hybrids, acidic and basic sites coexistence, tailored synthesis, mesoporous silica based hybrids, physico-chemical characterization, probe molecules, multi-technique characterization

## Abstract

The development of organic–inorganic hybrid materials bearing both acidic and basic functionalities is a challenging task, as it requires preserving the integrity and availability of two distinct active sites within a single porous network, such as silica. In this study, we report a synthetic approach that combines established grafting and co-condensation methods to achieve a controlled distribution of acidic and basic sites in mesoporous silica. Although each strategy is well known individually, their deliberate integration provides a distinctive pathway to tune the spatial arrangement and mutual preservation of the two functionalities. A comprehensive, multi-technique characterization approach, including in situ analyses and probe molecule adsorption, was used to monitor structural and chemical changes in both the organic and inorganic components. The results reveal that the coexistence of acidic and basic groups is highly sensitive to the synthetic conditions and activation treatments. In particular, basic sites are prone to protonation during the conversion of thiol groups into strong sulphonic acid sites, resulting in a partial loss of basic activity. The extent of this effect depends on the specific preparation route. In conclusion, the combined synthetic and characterization approach offers valuable insights into the nature, stability, and availability of the functional sites, guiding the rational design of advanced bifunctional hybrid materials.

## 1. Introduction

The design and development of multifunctional organic–inorganic hybrid materials represent a prominent topic in material chemistry due to their great versatility, making them highly attractive for a wide range of applications in the areas of heterogeneous catalysts, pollutant removal, medical diagnosis, therapy, and energy storage [1,2,3,4,5]. Among the various hybrid materials, in the last few decades, organic–inorganic porous hybrids have gained more scientific attention, especially in the field of heterogeneous catalysis, due to their high specific surface area, large pore volume, and tuneable and ordered porous architectures [6,7,8]. The porous structure of inorganic materials allows them to accommodate a wide variety of organocatalytic functionalities, providing active center isolation inside the inorganic architecture. Interestingly, it is possible to host both acidic and basic active sites within the same inorganic structure, even though they would neutralize each other when in solution [9,10]. Active sites in acid–base bifunctional hybrids can work together synergistically, providing superior reactivity compared to monofunctional materials. Moreover, these hybrids can promote complex reactions, including multistep or cascade processes [11,12]. Nevertheless, for such efficiency and synergy to be sustained, the acid and basic sites must preserve their intrinsic properties even after the various post-synthesis treatments applied to the hybrids. Furthermore, the growing need to manage catalyzed chemical reactions under milder conditions, such as lower temperatures, has led to the development of hybrid materials that combine the mechanical strength and thermal resistance properties of inorganic matrices with the wide variability of the organic components.

When designing a bifunctional porous hybrid material, different synthetic strategies should be considered to optimize the localization and availability of different organic functional sites inside the porous inorganic network. Typically, a single organic functional molecule, generally an organosilane, can be covalently grafted to the reactive surface groups of inorganic matrices, such as silanols in silica-based supports, via a post-synthetic grafting approach [2,8,13,14,15]. The preparation of bifunctional hybrid materials involves introducing a second organic moiety, which significantly increases the complexity of the synthetic process. This added complexity often leads to a non-uniform spatial distribution of the organic functional groups, decreasing the homogeneity of active site distribution within the hybrid material and ultimately limiting its practical application [16]. Furthermore, the dispersion of inorganic solids and organosilane compounds in grafting procedures is obtained by the use of organic solvents such as toluene [15,17] or more renewable alternatives, as recently reported [18]. An alternative strategy for the incorporation of two functional organic groups is the co-condensation method, also referred to as one-pot synthesis: in this case, the functional organic silanes are introduced in the synthesis gel during the formation of ordered mesoporous silica [9]. This methodology is often preferred because it relies on a relatively simple synthetic protocol, while allowing more precise control over the organic functional groups’ loading and typically ensuring better homogeneity in their spatial distribution. A critical limitation of this approach is associated with the removal of the surfactant employed as structure-directing agents (SDA) in the synthesis of ordered mesoporous silica. To address this issue, alternative template-extraction strategies, such as solvent extraction, are often required; nevertheless, these methods are generally less effective compared to the conventional high-temperature calcination treatment, which remains the most reliable route for achieving complete surfactant elimination [19,20].

Given that the two methodologies described above are widely used and their advantages and disadvantages have been extensively reviewed in the literature [8,9,16], and considering the inherent challenges associated with the simultaneous incorporation of two distinct organic functionalities into a single material, alternative synthetic strategies can be adopted for the preparation of bifunctional hybrid systems. These approaches offer enhanced control over the spatial distribution and availability of each functional group, which can significantly improve the overall performance of the hybrid. Furthermore, they provide fine tuning of the physico-chemical properties of the resulting hybrid systems, facilitating their optimization for targeted applications and broadening their usefulness across diverse fields. Introducing one functionality via the co-condensation method and the other one via the post-synthesis grafting would ideally lead to a large spatial separation since the first functionality would be homogeneously embedded in the material network, while the grafting methodology would primarily direct the second functionality on the material surface. Despite the advantages related to improved localization, some drawbacks inherited from combining two synthetic methodologies must be addressed, such as the presence of a multi-step procedure and the lower efficiency of surfactant removal through solvent extraction techniques, while maintaining the porous regularity of the material without deactivating the organic functions. In this regard, Soxhlet extraction in acidic environments and in rather extreme conditions (200 °C for 72 h) still remains the most used method for the removal of surfactant. By contrast, in this work, a mild sonication procedure was employed as a gentler, faster, and more sustainable approach for the removal of the SDA from the mesopores [20,21].

In the present work, bifunctional hybrid materials containing simultaneously acidic and basic sites within Ordered Mesoporous Silica Nanoparticles (OMSNs) were synthesized using three distinct methodologies: simultaneous post-synthetic grafting of the two organic silanes, co-condensation (one-pot), and a combined approach integrating grafting and co-condensation. A detailed investigation of these synthetic strategies was carried out to assess the distribution and availability of acidic and basic sites, with the aim of elucidating their influence on the overall structural and chemical properties of the materials. Such an evaluation is essential for optimizing site availability and spatial arrangement, which are key factors in the rational design of multifunctional hybrids with enhanced activity and selectivity [22].

OMSNs with a structure similar to ordered mesoporous MCM-41 were chosen as inorganic hosts due to their well-known properties as inorganic support, whilst (3-aminopropyl)triethoxysilane (APTS) and (3-mercaptopropyl)trimethoxysilane (MPTS) were chosen as the organic functionalities to introduce, respectively, basic sites (-NH_2_) and thiol groups as precursors of acid sites. In fact, the thiol groups can be subsequently oxidized to sulphonic acids (-SO_3_H), providing also acid properties to the hybrid materials [23]. Different synthesis parameters were explored, such as the loading and the addition order of the organic moieties. To understand the structure–property relationship of these hybrid materials in greater depth, a multi-technique characterization was performed by using X-ray powder diffraction (XRPD), thermogravimetric analysis (TGA), CHNS elemental analysis, and Fourier-Transform Infrared (FTIR) spectroscopy supplemented by the use of probe molecule. It is worth noting that, in these complex hybrid materials containing multiple chemical functionalities, a key challenge is to identify the individual functionalities to understand the nature of the active sites after the different treatments applied to the hybrids. Achieving this requires the use of combined techniques and the integration of the resulting information.

## 2. Materials and Methods

### 2.1. Chemicals

Ordered mesoporous hybrid materials were synthesized using tetraethyl orthosilicate (TEOS, Aldrich, St. Louis, MO, USA) as silica source, and (3-aminopropyl)-triethoxysilane (APTS, ≥98%, Aldrich) and (3-mercaptopropyl)-trimethoxysilane (MPTS, ≥95%, Aldrich) as organic functionalities. NaOH solution (NaOH, ≥97%, Aldrich) was used as the basifying agent of the sol-gel synthesis, and cetyltrimethylammoniumbromide (CTAB, ≥99%, Aldrich) as the mesoporous structure-directing agent (SDA). 2-Methyltetrahydrofuran (2-MeTHF, ≥99%, Carlo Erba, Cornaredo, Italy) was used as solvent in the post-synthetic organic grafting and 2-propanol (≥99.5%, Aldrich) was used as solvent for SDA removal. Hydrogen peroxide solution (H_2_O_2_, 30% *w*/*w*, Aldrich) was used for oxidation of thiol groups into sulphonic groups.

### 2.2. Synthesis of Ordered Mesoporous Silica Nanoparticles (OMSNs)

Ordered mesoporous silica nanoparticles (OMSNs) were synthesized following the procedure reported in ref. [24] using CTAB as a structure-directing agent and TEOS as a silicon source. CTAB (1 g) was dissolved in 480 mL of deionized water and an NaOH solution (2.0 M, 3.5 mL) was added at 80 °C. Subsequently, TEOS (5 mL) was added. The mixture was stirred at 80 °C for 2 h; the obtained solid product was filtered and washed with deionized water and ethanol and, afterwards, was calcined in air flow at 600 °C for 6 h with a ramp of 1.5 °C/min to remove the SDA.

### 2.3. Synthesis of Bifunctional Mesoporous Hybrids

The bifunctional mesoporous hybrids were synthesized following three different synthetic approaches (post-synthetic grafting, co-condensation, and a combined method integrating both grafting and co-condensation) with two different organic loadings.

### 2.4. Post-Synthetic Grafting of Organic Functionalities on OMSNs

The post-synthetic grafting procedure was carried out according to Brito et al. [18] by suspending the calcined OMSNs in 2-methyltetrahydrofuran (2-MeTHF) and adding the two organosilane, (3-mercaptopropyl)trimethoxysilane (MPTS) and (3-aminopropyl)triethoxysilane (APTS), keeping the solution under stirring at 60 °C for 4 h; the solid was then filtered and washed with deionized water and ethanol.

### 2.5. Co-Condensation Synthesis of Functionalized OMSNs

The co-condensation synthesis of the functionalized hybrids was performed following the procedure described for the synthesis of plain OMSNs by adding APTS and MPTS into the synthesis gel. After the drop-by-drop addition of TEOS, the solution was stirred for 20 min, allowing complete pre-hydrolysis of the siliceous source and the two organosilanes, MPTS and APTS, were subsequently added within 5 min of each other. The mixture was stirred at 80 °C for 2 h to give rise to a white precipitate. The solid product was filtered, washed with deionized water, and dried in an oven at 80 °C overnight. Afterwards, the template was removed by sonication procedures.

### 2.6. Grafting and Co-Condensation Combined Method of Functionalized OMSNs

The combined method involves co-condensation of one organic functionality followed by grafting of the second functionality. For each type of bifunctionalization carried out, the order of insertion of the organosilane precursors was reversed in order to observe any differences in the loading of the functionalities and, consequently, of the catalytic activity. Finally, the SDA was removed by sonication procedure.

### 2.7. SDA Removal Procedure

The ultrasound-assisted CTAB removal was carried out in an ultrasonic cleaner ARGOLAB (SINERGICA^®^, Milan, Italy) with an ultrasound power of 120 W and frequencies of 40 kHz at 40 °C for 20 min, according to the optimized conditions reported in ref. [20]. One gram of hybrid was dispersed in 6 mL of 2-propanol and immersed into water in the ultrasonic device. After ultrasonic irradiation, the solid was recovered by centrifugation. This procedure was repeated for ten times and finally dried at 80 °C overnight. The supernatants obtained from centrifugation were qualitatively analyzed by FT-IR spectroscopy after each extraction (Figure S1), confirming the presence of the surfactant in solution and consequently the correct extraction procedure. The ultrasonic templating removal procedure was performed for the hybrids synthesized through the co-condensation and mixed synthesis, while the SDA removal from OMSNs for the post-synthesis grafting was conducted by calcination at 600 °C.

### 2.8. Thiol Groups Oxidation

The oxidation procedure of the MPTS thiol groups to sulphonic groups was carried out with treatment with H_2_O_2_ solution 30% (*w*/*w*). For the oxidation, 1 g of hybrid was added over 30 mL of H_2_O_2_. The solution was left under stirring at room temperature for 5 h. The solid was recovered by centrifugation at 7000 rpm for 5 min and washed with distilled water to neutral pH.

### 2.9. Characterization Techniques

All the bifunctional hybrids were characterized by XRPD, thermogravimetric and CHNS elemental analysis, and FTIR spectroscopy using probe molecules to monitor the organic functionalities of the hybrids.

X-Ray powder diffraction (XRPD) analysis was carried using a Bruker AXS D8 ADVANCE diffractometer (Bruker AXS GmbH, Karlsruhe, Germany) in reflection mode with Bragg–Brentano geometry, operating with a radiation source of monochromatic X-rays Cu Kα (λ = 1.5406 Å), and an LYNXEYE_XE_T high-resolution position-sensitive detector. XRPD patterns were recorded in the 0–10° (2θ) range at the voltage and amperage of the source 40 kV/40 mA with a coupled 2θ-θ method at a scan speed of 0.100 s/step and a step size of 0.01°. Field Emission Scanning Electron Microscopy images were taken with a GeminiSEM-360 instrument (Carl Zeiss S.p.A., Milan, Italy). For the observation, samples were Pt-sputter-coated (4 nm thick Pt layer) under argon. The samples were imaged at acceleration voltages between 5 and 10 kV, using the InLens SE detector and working distances between 1.8 and 2.1 mm. Volumetric analyses (N_2_ adsorption/desorption isotherms at 77 K) were obtained with a Micromeritics ASATM 2420 (Micromeritics Instrument Corporation, Norcross, GA, USA), after a pretreatment at 100 °C for 2 h under vacuum. The specific surface areas (SSA) were calculated using the Brunauer–Emmett–Teller (BET) equation in the relative pressure range varying from 0.01 to 0.1 P/P_0_. The pore size distribution was calculated from the Barrett–Joyner–Halenda (BJH) method, using the desorption region of the N_2_ physisorption isotherm. Thermogravimetric and differential thermal analysis (TGA-DTA) were carried out in air stream (60 mL/min) using a Setaram LABSYS evo instrument (Setaram Instrumentation, Caluire-et-Cuire, France), heating from 30 to 800 °C at 5 °C/min. C–H–N-S elemental contents were determined using an EA 3000 elemental analyzer (EuroVector S.p.A., Pavia, Italy). Helium and oxygen at 120 and 35 kPa pressures were used, respectively. For each material, three measurements have been performed.

FT-IR spectra were obtained using a Nicolet 5700 (Thermo Fisher Scientific, Waltham, MA, USA) spectrometer with 64 scans and a resolution of 4 cm^−1^ in a 4000–400 cm^−1^ spectral range using self-supporting pellets. Spectra were collected after thermal treatment at 150 °C for 1 h under vacuum conditions (residual pressure < 10^−2^ mbar). FT-IR spectra were normalized with respect to the pellet density. The FT-IR spectroscopic study was implemented through the adsorption probe molecules that were able to detect the presence of active sites on the surface of the synthesized hybrids. The acid functionality related to the sulphonic group was detected using ammonia as a basic probe molecule.

## 3. Results and Discussion

**Scheme 1:**
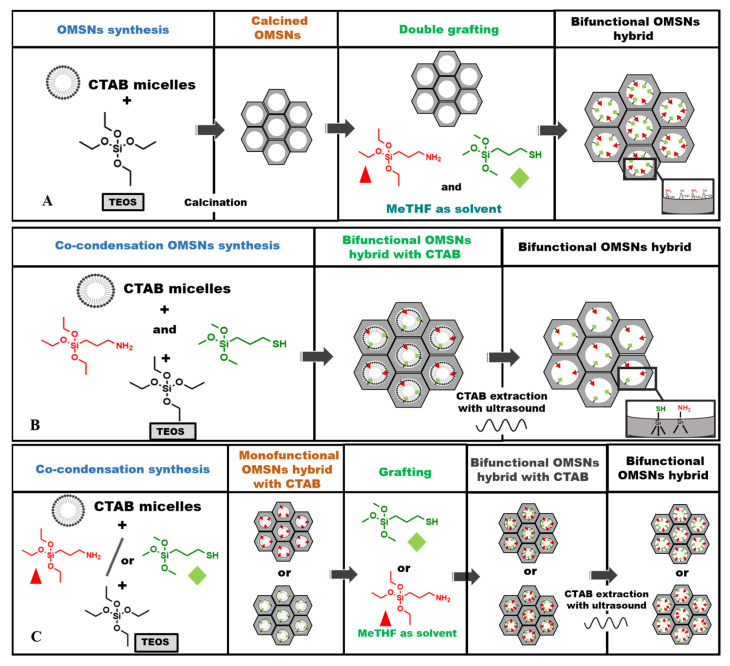
Bifunctional mesoporous hybrids synthesized by post-synthetic grafting (**A**), co-condensation (**B**), and mixed (grafting and co-condensation) method (**C**). Red triangles represent APTS; green diamonds represent MPTS.

Bifunctional organic–inorganic hybrids were developed using ordered mesoporous silica nanoparticles as the inorganic support, functionalized with APTS, to introduce basic sites and with MPTS as a precursor of acid sites. The thiol groups of MPTS were successively oxidized to sulphonic groups (–SO_3_H) using an H_2_O_2_ solution to obtain acid/base bifunctional hybrids. Among the various synthetic strategies to covalently bind organic functional groups into the mesoporous silica network, post-synthetic grafting is widely used. However, this method presents several drawbacks, including partial pore blockage and undesirable site–site interactions caused by the supramolecular association of silylated organic precursors. For these reasons, the co-condensation approach can be considered a valid alternative. It offers a simple and streamlined one-pot method to integrate organic functional groups directly within the inorganic silica network during the synthesis and allows for better control over organosilane loading, resulting in a more homogeneous distribution of active sites. When the incorporation of two distinct organic functionalities, such as acidic and basic sites, into a single inorganic material is required, the synthetic challenge becomes considerably more demanding. This complexity arises from the need to prevent mutual deactivation between the opposing functional groups, which can compromise their individual catalytic performance. To address this, it is crucial to ensure a well-defined separation between the acidic and basic sites, thereby preserving the intrinsic properties and reactivity of each active site within the hybrid network. In line with these considerations and to gain deeper insight into the dispersion and potential interactions between active sites of differing natures, three distinct synthetic approaches were investigated: post-synthetic grafting (Scheme 1A), co-condensation (Scheme 1B), and a combined method integrating both strategies (Scheme 1C), hereinafter referred to as the mixed procedure. These methods were evaluated to assess the feasibility of incorporating antagonistic acidic and basic functionalities within a single hybrid material. In both the grafting and co-condensation methods, APTS and MPTS were introduced simultaneously, whereas in the mixed procedures, two distinct hybrids were synthesized by varying the sequence of silane addition; this information is reflected in the acronyms assigned to the hybrids. Additionally, two distinct organic loadings, referred to as low loading and high loading, were evaluated to assess their impact on the successful integration and distribution of these functionalities [25,26]. The acronyms of the hybrids upon oxidation of thiol groups, together with the organic content of APTS and MPTS, are reported in Table 1.

**Figure 1:**
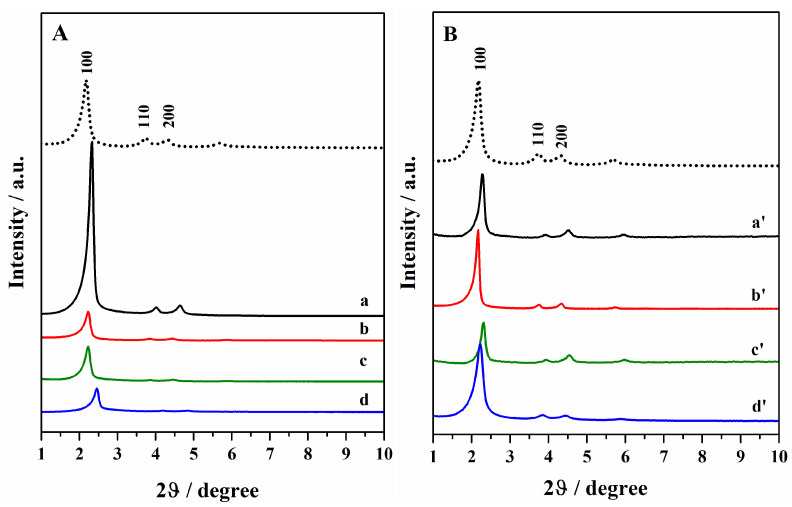
Powder XRD patterns of hybrids after the template removal by sonication and oxidation of thiol groups. (**A**): H_gr_-NH_2_+SO_3_H @OMSNs (curve a), H_mix_-NH_2_+SO_3_H @OMSNs (curve b), H_mix_-SO_3_H+NH_2_@OMSNs (curve c), H_co_-NH_2_+SO_3_H @OMSNs (curve d) and (**B**): L_gr_-NH_2_+SO_3_H@OMSNs (curve a′), L_mix_-NH_2_+SO_3_H@OMSNs (curve b′), L_mix_-SO_3_H+NH_2_@OMSNs (curve c′), and L_co_-NH_2_+SO_3_H @OMSNs (curve d′). In (**A**), the XRD pattern of calcined OMSNs is also reported (dotted curve).

The ordered mesoporous hybrids were characterized by XRPD analysis (Figure 1) after the template removal and the oxidation of thiols to sulphonic groups. The diffraction pattern of the hybrids mainly exhibited the (100) reflection peak at low diffraction angles, which is characteristic of ordered hexagonal mesostructured (p6 mm symmetry) porous silica materials such as MCM-41 [27,28]. In some bifunctional hybrids, especially in those obtained through double grafting (curve a and a′), the peaks at higher diffraction angles (110 and 200) are also visible, confirming the long-range periodic order of the mesopores. However, the reduced intensity of these peaks in the other hybrids indicates a loss of the long-range order of the mesoporous structure, due to the organic moieties’ insertion inside the silica network during the co-condensation (curve d and d′) and mixed synthesis routes (curves b, b′, c, and c′).

To evaluate the effect of the synthetic approach on particle morphology, FE-SEM analysis was carried out (Figure S2). The images reveal that the three hybrid materials exhibit some differences in morphology and particle uniformity. Samples prepared by grafting (Figure S2A) and by co-condensation (Figure S2B) display a comparable spherical morphology, with relatively homogeneous particle size distributions. On the other hand, the sample obtained through the mixed procedure (Figure S2C) shows a less regular morphology, characterized by slightly elongated, aggregated, and non-spherical particles with sizes exceeding 200 nm. This morphological difference may be attributed to the mixed synthetic procedure, which leads to partial aggregation and resulting in the irregular and elongated structures observed.

The textural properties of the bifunctional hybrids, determined by volumetric analyses (N_2_ adsorption/desorption isotherms at 77 K), are characteristic of ordered mesoporous silicas. Compared to the calcined OMSNs, all hybrids exhibit a decrease in specific surface area, pore size, and pore volume due to the functionalization of the inorganic support with organosilanes (Table S1 in the Supplementary Materials). In particular, the hybrid obtained by post-synthetic grafting shows a specific surface area (SSA) of 580 m^2^ g^−1^, whereas the hybrid prepared by co-condensation displays a much more pronounced reduction, likely due to the presence of residual SDA within the mesopores.

Elemental and thermogravimetric analyses were carried out on the hybrids after thiol oxidation to provide a detailed assessment of the organic content in the hybrid materials. These analyses not only allowed evaluation of the efficiency of cationic surfactant (CTAB) removal through sonication but also confirmed the successful introduction of organic functional groups. Importantly, the results also highlighted the thermal stability of the organic part of the synthesized hybrids.

The elemental composition, including carbon (C), hydrogen (H), nitrogen (N), and sulfur (S), are summarized in Table 2. Bifunctional mesoporous hybrids prepared via the co-condensation and mixed methods showed higher organic content than those obtained by grafting, which is mainly attributed to residual CTAB, as confirmed by thermogravimetric analysis. Surfactant removal by sonication reached an efficiency of approximately 70%, which is comparable to that achieved with Soxhlet extraction (Table S2 in the Supplementary Materials), but obtained under substantially milder conditions, in terms of both processing time and temperature.

**Figure 2:**
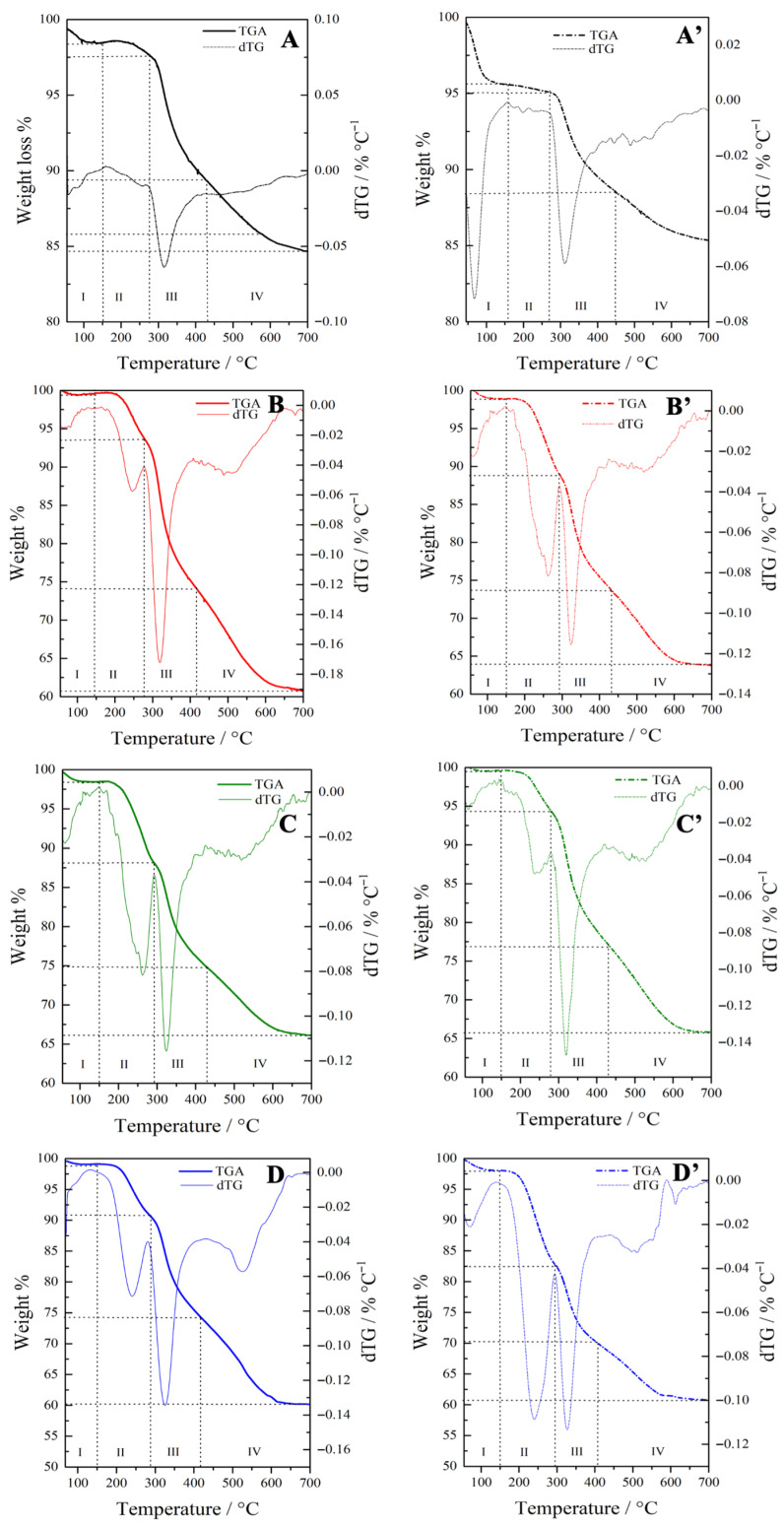
TGA and dTG analysis of (**A**) H_gr_-NH_2_+SO_3_H@OMSNs, (**A′**) L_gr_-NH_2_+SO_3_H@OMSNs, (**B**) H_mix_-NH_2_+SO_3_H@OMSNs, (**B′**) L_mix_-NH_2_+SO_3_H@OMSNs, (**C**) H_mix_-SO_3_H+NH_2_@OMSNs, (**C′**) L_mix_-SO_3_H+NH_2_@OMSNs, (**D**) H_co_-NH_2_+SO_3_H@OMSNs, and (**D′**) L_co_-NH_2_+SO_3_H@OMSNs.

Thermogravimetric analysis was carried out to quantify the contributions of CTAB and the organic groups by monitoring the corresponding weight losses (Figure 2). For comparison, the TGA profiles of pure CTAB and CTAB-containing OMSNs were also considered (Figure S3). The weight loss percentages of all hybrids across different temperature ranges are summarized in Table 3.

The bifunctional hybrids displayed four main weight loss regions. The first region (zone I), below 150 °C, corresponds to the desorption of physisorbed water. In general, high-loading hybrids exhibit reduced hydrophilicity, as many of the silanol groups are involved in interactions with the organic functionalities. Furthermore, hybrids obtained either through co-condensation or via the mixed procedure consistently display a lower amount of physisorbed water, suggesting that the synthetic pathway plays a critical role in modulating surface water affinity. The grafted silanes start to decompose at a temperature higher than 280 °C (Figure 2A,A′). The second region (zone II), observed between approximately 150 °C and 300 °C, is mainly associated with the removal of the CTAB from the mesoporous silica framework in hybrids obtained via co-condensation or mixed procedures (Figure 2B,B′,C,C′,D,D′). This interpretation is consistent with the TGA profiles of pure CTAB and CTAB-containing OMSNs (Figure S3A,B). In this regard, sonication proved effective in extracting the surfactant, as indicated by the lower weight loss in zone II compared to CTAB-containing OMSNs (Figure S3B, Table 3). Conversely, in hybrids prepared by grafting, which are free of CTAB, the organic groups linked to the grafted silanes begin to decompose only at temperatures above 280 °C (Figure 2A,A′). The third weight loss region (zone III) between 300 and 400 °C, can be mainly ascribed to the degradation of organic functional groups originating from the organosilanes. This assignment is supported by the TGA profiles of previously calcined bifunctional mesoporous hybrids obtained via the grafting method (Figure 2A,A’), which exhibited negligible surfactant content. At temperatures above 400 °C (zone IV), the weight loss is mainly attributed to the decomposition of APTS/MPTS-derived organic moieties, which has a greater contribution than the dehydroxylation of surface silanols (Figure S4).

The oxidation of thiol groups into sulphonic acids, which allows the coexistence of acidic and basic active sites within the same material, is a key step in the development of bifunctional hybrids. To better elucidate the potential interactions between these two opposing organic functionalities, a thorough physico-chemical characterization is required. FTIR spectroscopy combined with the use of probe molecules provides valuable insight into both the nature and the availability of the active sites. Prior to FTIR measurements, all samples were outgassed at 150 °C for 2 h to eliminate physisorbed water.

**Figure 3:**
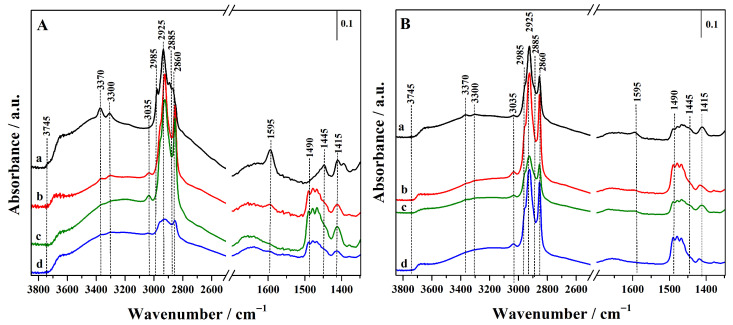
FTIR spectra of the synthesized hybrid materials before oxidation step. (**A**): H_gr_-NH_2_+SH@OMSNs (curve a), H_mix_-NH_2_+SH@OMSNs (curve b), H_mix_-SH+NH_2_@OMSNs (curve c), H_co_-NH_2_+SH@OMSNs (curve d) and (**B**): L_gr_-NH_2_+SH@OMSNs (curve a), L_mix_-SH+NH_2_@OMSNs (curve b), L_mix_-SH+NH_2_@OMSNs (curve c), and L_co_-NH_2_+SH@OMSNs (curve d).

FTIR spectra of all hybrids before thiol oxidation are reported in Figure 3, and the acronyms have been revised to emphasize the presence of thiol (–SH) rather than sulphonic (–SO_3_H) groups.

In the high-wavenumber region, no bands attributable to the O–H stretching vibrations of free surface silanol groups (≈3745 cm^−1^) are visible. This absence suggests that all isolated silanol groups were consumed during the introduction of organic functionalities, also in the hybrid with low organic content (Figure 3B). A broad adsorption between 3700 and 3200 cm^−1^ is also visible, due to the silanols interacting via H-bond that are always present on the silica surface [29]. In this region, bands at 3370 and 3300 cm^−1^, assigned to the asymmetric and symmetric N–H stretching modes of amine (–NH_2_) groups from APTS, are also observed, together with the band at 1595 cm^−1^, attributed to the scissoring bending mode of NH_2_ groups, confirming the successful functionalization of silica nanoparticles with APTS. This latter feature is particularly pronounced in samples with higher silane content (Figure 3A) and in the hybrids prepared via grafting (curve a) and mixed procedure by adding APTS in the synthetic gel and grafting MPTS (curves b). Moreover, the asymmetric and symmetric stretching modes for methyl (2985 and 2885 cm^−1^) and methylene (2925 and 2860 cm^−1^) groups of the propyl chains of organosilanes (APTS and MPTS) and residual CTAB species are detected, together with the corresponding bending modes observed between 1490 and 1445 cm^−1^ and at 1415 cm^−1^, respectively [14,30,31].

The higher intensity of these latter bands in the hybrids prepared via co-condensation and mixed procedures (curves b, c and d) is due to the presence of residual surfactant molecules. This interpretation is further supported by a signal at 3035 cm^−1^, assigned to methyl groups bound to the quaternary nitrogen of CTAB. In contrast, this signal is absent in hybrids obtained via the double-grafting method (curves a), where CTAB is completely removed by calcination at high temperature.

**Figure 4:**
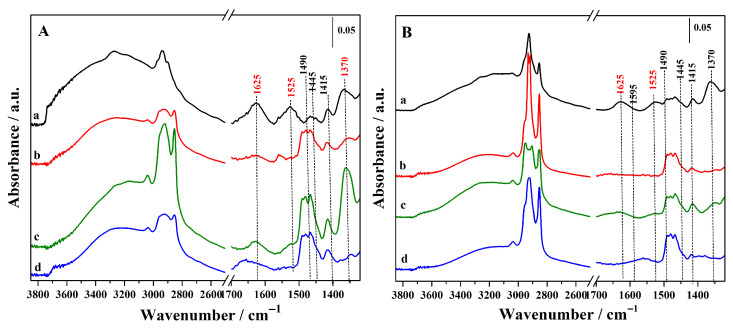
FTIR spectra of the synthesized hybrid materials after oxidation step. (**A**): H_gr_-NH_2_+SO_3_H@OMSNs (curve a), H_mix_-NH_2_+SO_3_H@OMSNs (curve b), H_mix_-SO_3_H+NH_2_@OMSNs (curve c), H_co_-NH_2_+SO_3_H@OMSNs (curve d) and (**B**): L_gr_-NH_2_+SO_3_H@OMSNs (curve a), L_mix_-SO_3_H+NH_2_@OMSNs (curve b), L_mix_-SO_3_H+NH_2_@OMSNs (curve c) and L_co_-NH_2_+SO_3_H@OMSNs (curve d).

Following the confirmation of the primary organic functionality, the amino groups (–NH_2_) in Figure 3A,B, the presence of the secondary acidic functionality (–SO_3_H) was verified after the conversion of thiols into sulphonic groups upon oxidation with a H_2_O_2_ solution (Figure 4A,B). The successful formation of sulphonic groups was confirmed by the emergence of a new band at 1370 cm^−1^, corresponding to the S=O asymmetric stretching mode, which was consistently observed, albeit with variations in intensity depending on the organic loading, in all the oxidized hybrids [10,32]. In particular, this signal is more intense in the sample H_mix_-SO_3_H+NH_2_@OMSNs (curve c, Figure 4A) prepared with the mixed procedure by adding MPTS in the synthetic gel and grafting APTS (curve c). For the hybrids prepared using the mixed procedure, FTIR analysis indicates that the organosilane introduced by co-condensation is present in greater amounts compared to the organosilane introduced by grafting within the same hybrid. This difference is likely due to the lower number of silanol groups available on the silica surface, which serve as the anchoring sites for grafting.

Notably, additional absorption bands emerge at 1625 and 1525 cm^−1^, which are attributed to the asymmetric and symmetric bending modes of protonated amino groups (–NH_3_^+^) [31]. Concurrently, the disappearance of the characteristic –NH_2_ bending band at 1595 cm^−1^ indicates a substantial transformation of the amino functionalities. This protonation process is most plausibly initiated during the oxidation step via proton transfer from H_2_O_2_. The absence of the NH_2_ vibrational features at 3373, 3305, and 1595 cm^−1^ (ν_sym_, ν_as_, and δ_as_) further corroborates that a large fraction of the amino groups has been protonated. Such modifications of the amino sites are expected to markedly impact the catalytic performance of these hybrids, particularly in reactions where basic functionalities play a pivotal role.

In all the investigated hybrid materials, it was consistently observed that the -NH_2_ basic sites of APTS are significantly affected by the oxidation treatment of the MPTS thiol groups required to generate the sulphonic acid centers. This phenomenon is not limited to materials obtained by post-synthetic grafting modification but is also evident in hybrids prepared via the co-condensation and mixed routes, in which the amino groups are less accessible to the external environment and therefore more effectively shielded, as they are embedded within the three-dimensional silica network. This evidence highlights the delicate balance between the generation of sulphonic acid sites through oxidative treatments and the preservation of amino basic functionalities, which are simultaneously desired in bifunctional hybrids. This finding is particularly relevant considering that APTS is among the most widely employed silane precursors for the introduction of basic functionalities into silica-based systems [33,34].

**Figure 5:**
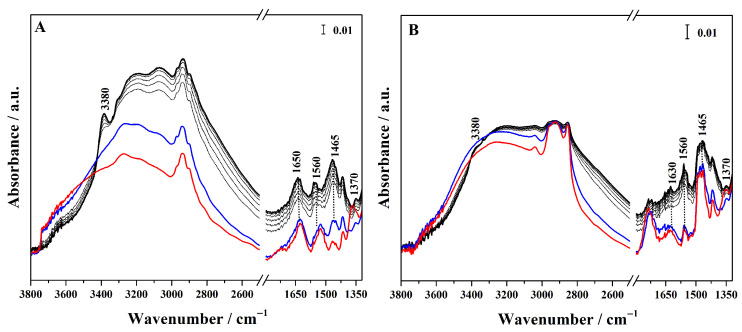

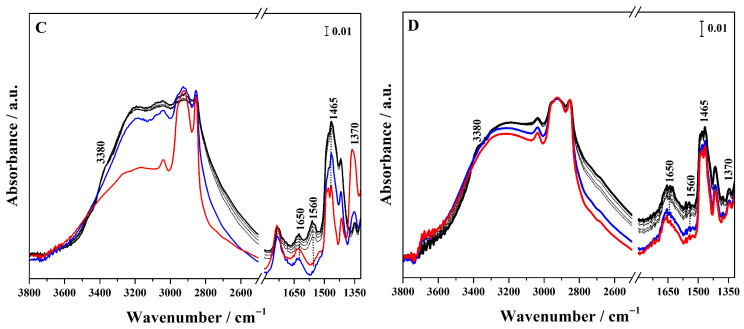
FTIR spectra of NH_3_ adsorption (maximum pressure 40 mbar, black curves) and desorption (gray curves) at 298 K on H_gr_-NH_2_+SO_3_H@OMSNs (**A**), H_mix_-NH_2_+SO_3_H@OMSNs (**B**), H_mix_-SO_3_H+NH_2_@OMSNs (**C**), and H_co_-NH_2_+SO_3_H@OMSNs (**D**) hybrids. Red curves correspond to the spectra recorded prior to NH_3_ adsorption, while blue curves correspond to the spectra after NH_3_ outgassing at 298 K. All hybrids were pretreated by outgassing at 150 °C for 1 h under vacuum before NH_3_ adsorption.

Conversely, to assess whether the sulphonic groups are available and protonated, and therefore able to act as Brønsted acid sites, ammonia was used as a sensitive probe molecule and its adsorption on the different hybrids was followed by FTIR spectroscopy (Figure 5 for high-loading and Figure S5 for low-loading hybrids). In particular, in the FTIR spectra of the grafted hybrid (Figure 5A), ammonia adsorption at high pressure (40 mbar, black curve) gives rise to signals at 3400 and 3320 cm^−1,^ corresponding to the N–H asymmetric and symmetric stretching modes of NH_3_ adsorbed via H-bond on surface silanols. These bands overlap with a broad feature cantered at 3020 cm^−1^ due to the O-H stretching mode of the silanols hydrogen-bonded to NH_3_. At a lower wavenumber, a band at 1650 cm^−1^ appears assigned to the asymmetric bending mode of the NH_3_ interacting with Si–OH groups. These spectroscopic features progressively decreased as the NH_3_ pressure is reduced (gray curves). The interaction of sulphonic groups with NH_3_ molecules results in the emergence of a band at 1465 cm^−1^, assigned to the asymmetric bending mode of NH_4_^+^ species, together with the concomitant disappearance of the sulphonic group band at 1370 cm^−1^. This spectroscopic evidence demonstrates that the sulphonic acid groups produced by the thiol oxidation exhibit sufficient Brønsted acidity to protonate ammonia [35,36]. The band assigned to NH_4_^+^ protonated species is also present in the hybrids synthesized through mixed routes (Figure 5B,C), but it is particularly pronounced in the FTIR spectra of H_mix_-SO_3_H+NH_2_@OMSNs (Figure 5C), suggesting greater accessibility of the acid sites. In contrast, in the hybrid obtained by co-condensation, this band appears with very low intensity, revealing the limited accessibility of the acid sites. These findings not only confirm the presence of Brønsted acid sites but also demonstrate that their availability is strongly dependent on the synthetic strategy adopted.

To evaluate if residual NH_2_ groups are still present after thiol oxidation, CO_2_ was used as a probe molecule (Figure S6 in the Supplementary Materials). In the region below 1700 cm^−1^, irreversible bands characteristic of alkylammonium carbamate species are observed at 1630 and 1495 cm^−1^, corresponding to the bending vibrations of protonated NH_3_^+^ species, and at 1555 and 1430 cm^−1^, which are assigned to the asymmetric and symmetric stretching modes of COO^−^ group, respectively [37,38]. These bands indicate that, although most amino groups are protonated, some sites remain available for interaction with CO_2_.

## 4. Conclusions

The preparation of bifunctional organic–inorganic hybrids represents both an opportunity and a challenge, as the choice of the synthetic strategy is crucial to ensure the effective coexistence of the different functional groups. An inappropriate combination of synthetic steps or conditions may lead to undesired interactions between the acidic and basic moieties, resulting in mutual deactivation and a significant loss of functional performance. In this study, we demonstrated that in acid–base hybrids, the basic sites are particularly prone to protonation during the conversion of thiol groups into strong sulfonic acid sites, leading to a reduction in their intrinsic basic activity. To explore whether this deactivation phenomenon could be mitigated, several synthetic routes were investigated, revealing that its extent strongly depends on the specific preparation conditions. An integrated approach combining advanced synthesis with in situ characterization techniques, supported by probe molecule adsorption, emerges as a powerful method to elucidate the nature and availability of the active sites and to guide the rational design of next-generation bifunctional hybrids. Importantly, beyond the ability to introduce distinct organic functionalities with acidic or basic character either within or onto mesoporous silica frameworks, it is equally essential to assess their state and stability after activation treatments to ensure that the designed properties are effectively maintained.

## Figures and Tables

**Table 1:** Acronyms of the bifunctional hybrid materials synthesized.

Sample Acronym ^1^	TEOS:APTS:MPTS Ratio [mol/mol]	Synthetic Procedure
H_gr_-NH_2_+SO_3_H@OMSNs	1:0.16:0.16	Grafting
H_co_-NH_2_+SO_3_H@OMSNs	1:0.16:0.16	Co-condensation
H_mix_-NH_2_+SO_3_H@OMSNs	1:0.16:0.16	Mixed (APTS by co-condensation and MPTS by grafting)
H_mix_-SO_3_H+NH_2_@OMSNs	1:0.16:0.16	Mixed (MPTS by co-condensation and APTS by grafting)
L_gr_-NH_2_+SO_3_H@OMSNs	1:0.06:0.06	Grafting
L_co_-NH_2_+SO_3_H@OMSNs	1:0.06:0.06	Co-condensation
L_mix_-NH_2_+SO_3_H@OMSNs	1:0.06:0.06	Mixed (APTS by co-condensation and MPTS by grafting)
L_mix_-SO_3_H+NH_2_@OMSNs	1:0.06:0.06	Mixed (MPTS by co-condensation and APTS by grafting)

^1^ H = high loading; L = low loading; the synthesis procedure is reported in the subscript.

**Table 2:** Weight percentages (% *w*/*w*) of carbon, hydrogen, nitrogen, and sulfur, as well as the organic content (% *w*/*w*), estimated by CHN analysis of the bifunctional mesoporous hybrids.

Hybrids	%C	%H	%N	%S	%Organic Content
H_gr_-NH_2_+SO_3_H@OMSNs	6.93	2.70	1.46	3.08	14.17
L_gr_-NH_2_+SO_3_H@OMSNs	3.78	2.00	0.94	2.47	9.19
H_co_-NH_2_+SO_3_H@OMSNs	24.56	5.04	1.72	3.49	34.81
L_co_-NH_2_+SO_3_H@OMSNs	26.11	4.93	1.80	1.40	34.24
H_mix_-NH_2_+SO_3_H@OMSNs	24.55	4.96	1.75	2.90	34.16
L_mix_-NH_2_+SO_3_H@OMSNs	26.81	5.23	1.69	1.94	35.67
H_mix_-SO_3_H+NH_2_@OMSNs	23.43	4.82	2.00	2.98	33.23
L_mix_-SO_3_H+NH_2_@OMSNs	27.71	5.36	1.83	1.13	36.03

**Table 3:** Weight loss % of bifunctional mesoporous hybrids obtained by TGA in different temperature ranges.

Weight Loss, Δwt %				
Hybrids	Region I	Region II	Region III	Region IV
H_gr_-NH_2_+SO_3_H@OMSNs	1.60	1.00	8.10	4.66
L_gr_-NH_2_+SO_3_H@OMSNs	4.40	0.62	6.45	3.11
H_co_-NH_2_+SO_3_H@OMSNs	0.90	8.35	16.53	14.09
L_co_-NH_2_+SO_3_H@OMSNs	2.05	15.56	12.26	9.56
H_mix_-NH_2_+SO_3_H@OMSNs	0.56	5.90	19.45	13.22
L_mix_-NH_2_+SO_3_H@OMSNs	1.18	9.96	15.25	9.58
H_mix_-SO_3_H+NH_2_@OMSNs	1.49	10.43	13.37	8.67
L_mix_-SO_3_H+NH_2_@OMSNs	0.94	5.30	17.57	11.13
CTAB	-	85.87	9.41	-
CTAB-containing OMSNs	3.41	33.84	1.75	4.69

## Data Availability

The original contributions presented in this study are included in the article/Supplementary Materials. Further inquiries can be directed to the corresponding author.

## Supplementary Materials

The following supporting information can be downloaded at: https://www.mdpi.com/article/10.3390/ma18214937/s1, Figure S1: FT-IR spectra of the supernatant solutions collected after each extraction cycle in isopropanol under ultrasonic conditions Figure S2: FE-SEM images of the bifunctional hybrids; Figure S3: TGA and dTG analysis of CTAB and CTAB- containing OMSNs; Figure S4: Thermogram of calcined bare OMSNs; Figure S5: FTIR spectra of NH3 adsorption at 298 K on hybrid materials; Figure S6: FTIR spectra of CO_2_ adsorption at 298 K on Hgr-NH2+SO3H@OMSNs; Table S1: Textural properties of the hybrid materials; Table S2: Organic content in the calcined OMSNs and after the CTAB removal using Soxhlet or ultrasound (US) extraction estimated by elemental analysis.
