# Supplementary material for: Impact of the Synthetic Strategy on the Structure and Availability of Active Sites in Bifunctional Mesoporous Organic–Inorganic Hybrids
Source: Materials (Basel). 2025 Oct 29;18(21):4937. doi: 10.3390/ma18214937 (PMC12608969; doi:10.3390/ma18214937)
Supplement: Supplementary file 1 [file materials-18-04937-s001.zip › materials-3925468-supplementary.pdf]

Supporting Information

Impact of the synthetic strategy on the structure and availability of active sites in bifunctional mesoporous organic–inorganic hybrids

Julio Cesar Fernandes P. Brito [1], Gioele Ancora [2], Ivana Miletto [3] and Enrica Gianotti [1,*]

1 Department for Sustainable Development and Ecological Transition, Università del Piemonte Orientale, Piazza Sant'Eusebio 5, I-13100, Vercelli, Italy; juliocesar.fernandes@uniupo.it; enrica.gianotti@uniupo.it
2 Department of Science and Technological Innovation, Università del Piemonte Orientale, Viale Teresa Michel 11, I-15100, Alessandria, Italy; gioele.ancora@uniupo.it
3 Department of Pharmaceutical Sciences, Università del Piemonte Orientale, Largo Guido Donegani 2-3, I-28100, Novara, Italy; ivana.miletto@uniupo.it

* Correspondence: enrica.gianotti@uniupo.it

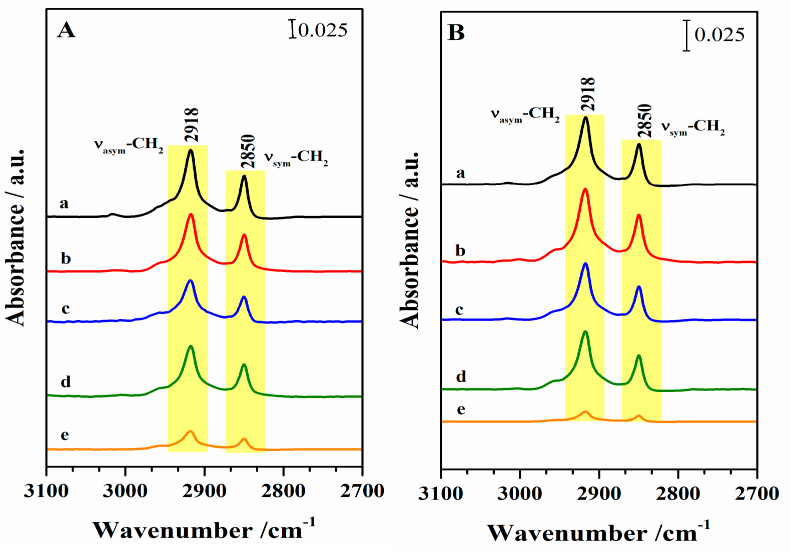

Figure S1. FT-IR spectra of the supernatant solutions collected after each extraction cycle in isopropanol under ultrasonic conditions for the materials synthesized via (A) the one-pot procedure and (B) the mixed procedure. The spectra correspond to the following: (a) 1° extraction cycle (black), (b) 2° cycle (red), (c) 3° cycle (blue), (d) 4° cycle (green), and (e) 5° cycle (orange). The progressive decrease in the intensity of the characteristic CTAB bands clearly indicates the gradual removal of the surfactant during the extraction process.

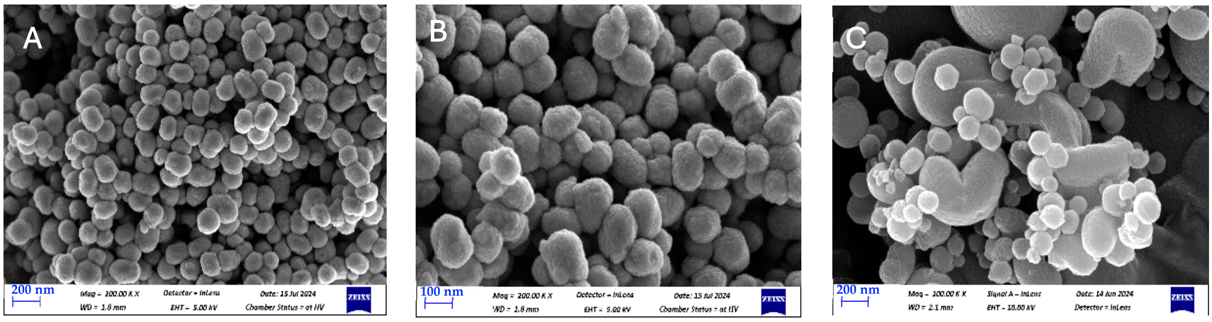

Figure S2. FE-SEM images of the bifunctional hybrids: H_{gr}-NH_2+SO_3H@OMSNs (**A**), H_{co}-NH_2+SO_3H@OMSNs (**B**), and H_{mix}-NH_2+SO_3H@OMSNs (**C**).

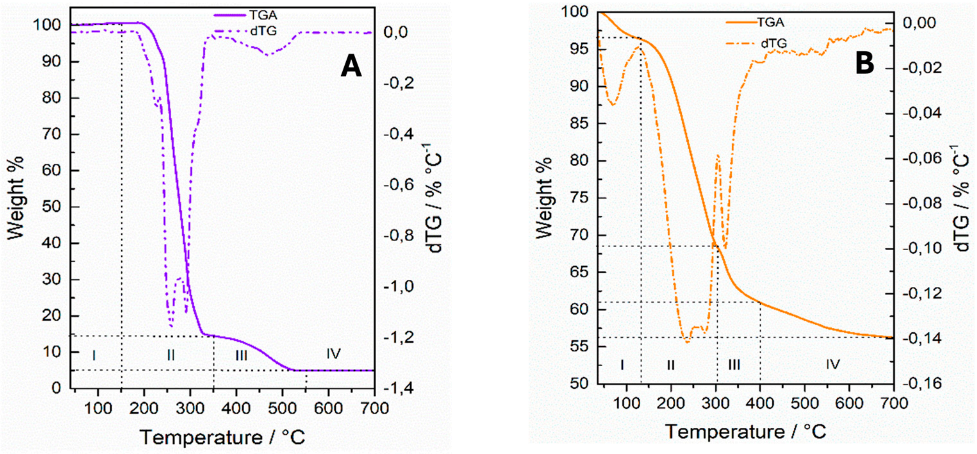

Figure S3. TGA and dTG analysis of CTAB (A) and CTAB-containing OMSNs (B).

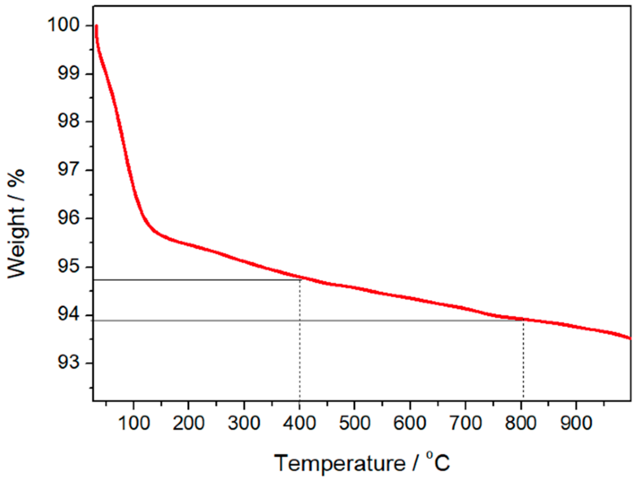

Figure S4. Thermogram of calcined bare OMSNs.

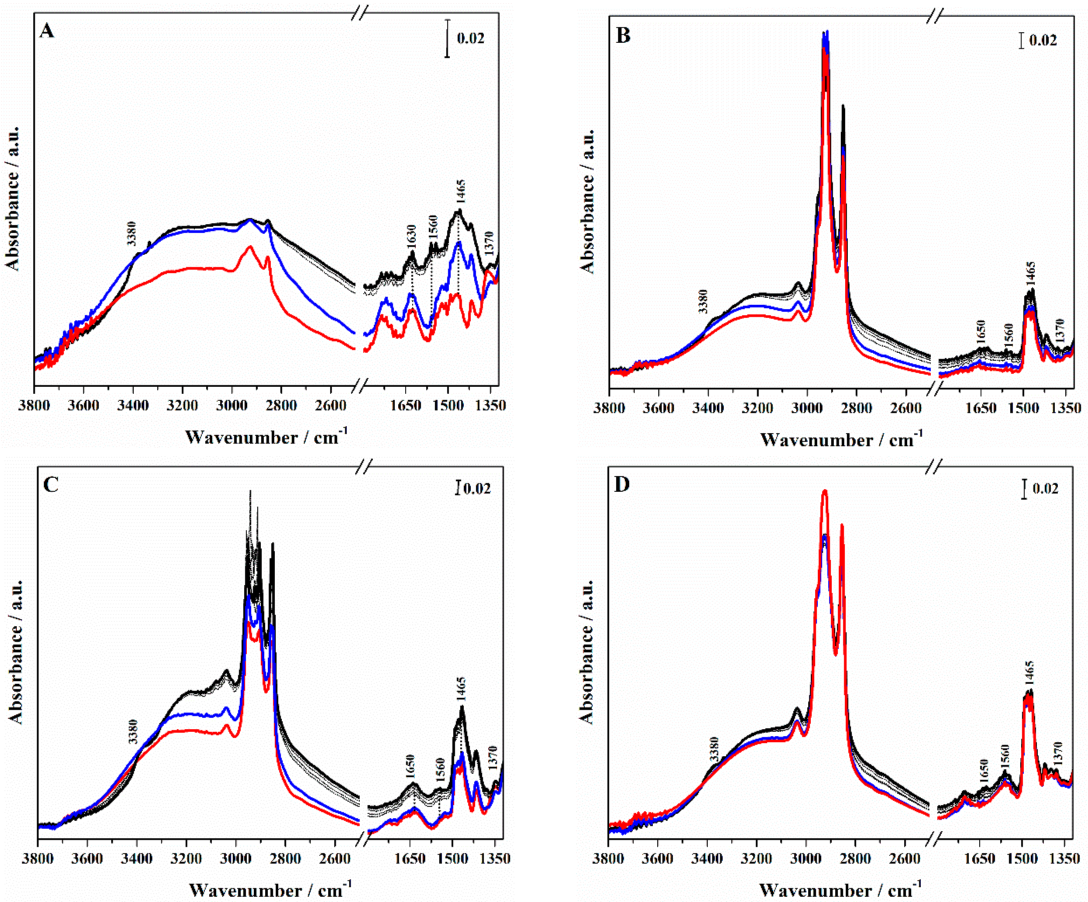

Figure S5. FTIR spectra of NH_3 adsorption (maximum pressure 40 mbar, black curves) and desorption (grey curves) at 298K on L_{gr}-NH_2+SO_3H@OMSNs (A), L_{mix}-NH_2+SO_3H@OMSNs (B), L_{mix}-SO_3H+NH_2@OMSNs (C), L_{co}-NH_2+SO_3H@OMSNs (D) hybrids. Red curves correspond to the spectra recorded prior to NH_3 adsorption, while blue curves correspond to the spectra after NH_3 outgassing at 298 K. All hybrids were pretreated by outgassing at 423 K for 1 h under vacuum before NH_3 adsorption.

Table S1 – Textural properties of the hybrid materials.

**Hybrids**	**SSA_{BET} ($m^2\ g^{-1}$)**	**d_{pores} (Å)**	**V ($cm^3\ g^{-1}$)**
Calcined OMSNs	1250	38	1.5
H_{gr}-NH_2+SO_3H@OMSNs	598	35	0.53
H_{co}-NH_2+SO_3H@OMSNs	150	34	0.13

Table S2 - Organic content in the calcined OMSNs and after the CTAB removal using Soxhlet or ultrasound (US) extraction estimated by elemental analysis.

Element %	Calcined OMSNs	OMSNs after Soxhlet extraction	OMSNs after US extraction
%C	0.13	26.45	29.55
%N	0	1.57	1.74
%H	1.18	5.58	6.15
%CTAB	1.32	33.61	37.44

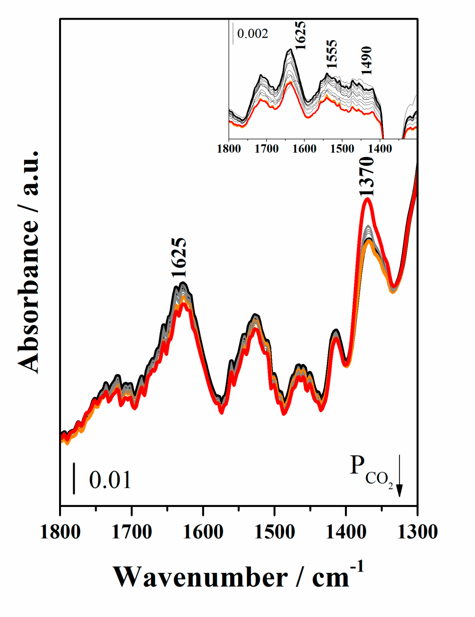

Figure S6. FTIR spectra, in the low wavenumber range, of CO_2 adsorption (maximum pressure 40 mbar, black curves) and desorption (orange curves) at 298 K on H_{gr}-NH_2+SO_3H@OMSNs. Inset: FTIR difference spectra.
